# My burning issues in neuroendocrine tumours (NET)

**DOI:** 10.1007/s12254-018-0449-2

**Published:** 2018-11-08

**Authors:** Barbara Kiesewetter, Markus Raderer

**Affiliations:** 1 Department of Internal Medicine I, Division of Oncology, Medical University Vienna, Waehringer Guertel 18–20, 1090 Vienna, Austria; 2 Neuroendocrine Tumour Unit, Comprehensive Cancer Center Vienna, Medical University Vienna, Vienna, Austria

**Keywords:** Somatostatin analogues, mTOR inhibitor, Everolimus, Gastro-enteropancreatic neuroendocrine tumor, Peptide receptor radionuclide therapy

## Abstract

Several compounds have recently been approved for the systemic treatment of advanced well-differentiated neuroendocrine tumours (NET) of gastroenteropancreatic (GEP) or lung origin. Based on the PROMID and CLARINET trials, somatostatin analogues (SSA) are the preferred first-line approach for all GEP-NET and offer—in addition to antiproliferative effects—durable symptomatic relief for hormonally active tumours. The mTOR inhibitor everolimus has been approved for progressive GEP- and lung-NET and is a widely used drug in this setting. Furthermore, recent results have underlined the high efficacy of somatostatin-receptor targeting radionuclide therapy (PRRT) in somatostatin-receptor positive midgut tumours and PRRT is now considered standard treatment for midgut-NET progressing on SSA. The optimal application of PRRT in somatostatin receptor positive NET with non-midgut site is currently an issue of discussion and should be decided on an individually basis in multidisciplinary boards. Following new insights in the genetic landscape of NET, “hot topics” in recent months include optimal treatment of the recently defined NET G3 and preliminary data on immunotherapy.

## Nomenclature in NEN—from classification to clinic

Neuroendocrine neoplasms (NEN) constitute a heterogeneous group of malignancies linked by their common origin of diffuse neuroendocrine cells [1]. They can arise in virtually any organ but are most commonly documented in the gastroenteropancreatic (GEP) tract (65%) and the lung (25%). According to the latest WHO classification, NEN are classified by morphological aspects and proliferation rate, i. e. Ki67 index/mitotic count into well differentiated neuroendocrine tumours (NET) with a Ki67 index ≤20% (including G1/G2 GEP-NET and typical/atypical carcinoid of lung); and undifferentiated, highly proliferative tumours with a Ki67 > 20% termed as neuroendocrine carcinomas (NEC; see Table 1; [2]). In addition, the WHO 2017 classification of neuroendocrine pancreatic tumours has added a novel category referred to as “NET G3” addressing a group of patients with a high proliferation index but well differentiated morphology [3]. This acknowledges the clinical observation that some patients with G3 tumours have a more indolent clinical course correlated with a sustained endocrine differentiation on histological assessment [4]. Whereas this term is currently restricted to patients with pancreatic (p)NETs, it is likely that NET G3 tumours can also arise from other primary sites. This is of clinical relevance insofar, as G3 NEC were exclusively treated with platin-based chemotherapy, but G3 NETs appear to respond unsatisfactory to platins and are potentially objective to distinct treatment algorithms based on therapy of highly differentiated NETs [5].

**Table 1:** WHO Classification 2010 for neuroendocrine neoplasms of gastrointestinal and pancreatic origin [2]

WHO Grade	Mitotic Count^a^	Ki-67 Index %
G1 NET	<2	≤2%
G2 NET	2–20	3–20
G3 NEC	>20	>20

^a^per 10 high power field

## Systemic treatment strategies for advanced NET

In addition to synaptophysin and chromogranin A as universal markers for neuroendocrine cells, most NETs of GEP-origin, but to a much lesser extent lung NETs express somatostatin receptors (SSR) allowing SSR-based functional imaging (octreotide scintigraphy, Ga68-PET) and SSR-targeted treatment [1]. While somatostatin analogues (SSA) have originally been developed for anti-secretory treatment of functional NETs, early preclinical data have suggested antiproliferative effects via SSR-dependent inhibitory crosstalk on autocrine signalling and growth factor secretion [6]. Finally, in the late 2000s the PROMID trial (octreotide LAR 30 mg q28 vs placebo) and later the CLARINET trial (lanreotide LAR 120 mg q28 vs placebo) showed that SSA application is able to decelerate tumour growth [7, 8]. Both trials demonstrated a significant progression-free survival (PFS) benefit versus placebo and resulted in approval of SSA for advanced GEP-NET and NET of unknown origin. Of note is, however, that pNET patients were excluded in the PROMID trial, proposing lanreotide as favourite compound for this subgroup. In terms of lung-NET, SSA are increasingly used as first line treatment in typical and atypical carcinoid, but no randomized data are available to confirm this approach [9]. In addition, the high rate of SSR-negative patients on imaging should add a note of caution to the uncritical and unlicensed use of SSA in patients with lung NETs.

The mTOR inhibitor everolimus has extensively been studied within the RADIANT trials, leading to approval for progressive GEP-, lung- and unknown origin NET regardless of functional status [10, 11]. In the RADIANT4 trial, everolimus resulted in a median PFS of 11.0 months versus 3.9 months (HR [hazard ratio] 0.48, 95% CI [confidence interval] 0.35–0.67) in the placebo arm for GI-, lung- and unknown origin NET with no new safety flags. The use of mTOR inhibitors is supported by investigational models suggesting a pronounced influence of the rapamycin (mTOR) pathway in NET tumourgenesis. However, agents with broader activity in the mTOR pathway such as BEZ235 to potentially circumvent resistance mechanisms associated with everolimus have not resulted in enhanced activity [12]. See Table 2 for approved drugs in GEP-NET and respective trials.

**Table 2:** Summary of approved drugs and respective trials for GEP-NET

Study	Setting	Prim. EP	Outcome
Octreotide vs. Placebo (*PROMID*) Rinke 2009, JCO [7]	Midgut or unknown origin NET (non-functioning and functioning)	TTP	14.3 m vs. 6 m (HR 0.34, 95% CI 0.20–0.59)
Lanreotide vs. Placebo (*CLARINET*) Caplin 2014, NEJM [8]	Ki-67 < 10% enteropancreatic or unknown origin NET (non-functioning)	PFS	Not reached vs. 18 m (HR 0.47, 95% CI 0.30–0.73)
Everolimus vs. Placebo (*RADIANT-3*) Yao 2011, NEJM [10]	Progressive disease pancreatic NET	PFS	11 m vs. 4.6 m (HR 0.35, 95% CI 0.27–0.45)
Everolimus vs. Placebo (*RADIANT-4*) Yao 2016, Lancet [11]	Progressive disease lung or GI NET (non-functioning)	PFS	11 m vs. 3.9 m (HR 0.48, 95% CI 0.35–0.67)
Sunitinib vs. Placebo Raymond 2011, NEJM [21]	Progressive disease pancreatic NET	PFS	11.4 m vs. 5.5 m (HR 0.42, 95%CI 0.26–0.66)
Streptozotocin + Doxorubicin + Fluorouracil Kouvaraki 2004, JCO [22]	Pancreatic NET	PFS/OS	2-year PFS 41%/ 2-year OS 74%

*Prim EP* primary endpoint,* vs.* versus, *NET* neuroendocrine tumour, *TTP* time to progression, *m* months, *GI* gastrointestinal tract*, PFS* progression-free survival*, HR* hazard ratio*, CI* confidence interval*, OS* overall survival

## PRRT—a story of success?

Peptide receptor radionuclide therapy (PRRT) using SSA labelled with yttrium (Y)90 or lutetium 177 (Lu177) constitutes a further targeted treatment approach for SSR-positive NETs [13]. With a maximum irradiation range of 0.5–2 mm for Lu177 and 2–5 mm for Y90 this treatment specifically targets NET-cells with (at least in theory) limited or no damage to surrounding tissues. The NETTER1 trial published in 2017 reported the first prospective randomized data for 177LU-DOATATE and could show an impressive PFS benefit of 28.4 months for PRRT versus 8.4 months for high-dose SSA (HR 0.21, 95% CI 0.14–0.33) in midgut-NET (i. e. small bowel, appendix, caecum and ascending colon) progressive to standard SSA treatment [14, 15]. In the following, updates on favourable quality-of-life (QOL) data and overall survival trends have kicked off a real “PRRT-hype” in the NET community [15]. However, despite positive data in smaller series for non-midgut primary tumours (including first retrospective data on NET G3) with SSR expression, it appears too early to recommend PRRT up-front for all eligible patients, particularly in view of documented late side effects including AML, MDS and nephrotoxicity [13]. According to current guidelines the only validated early-algorithm position for PRRT is second line, SSA-progressive midgut NET while all other patients should be carefully selected based on individual decisions via multidisciplinary tumour boards. In addition, it has to be stated that in the NETTER trial, PRRT was combined with ongoing octreotide at the standard dose of 30 mg, and also the approved label for PRRT is thus such a combination approach [14].

## News for refractory carcinoid syndrome

Up to one third of tumours produce ectopic peptides and hormones and are classified as functioning tumours [1]. The most prominent symptom complex is the so-called “carcinoid syndrome” caused by serotonin overproduction of well-differentiated ileal-NETs and results in diarrhoea, flushing ± wheezing. Furthermore, the serotonin overload causes structural problems particularly to the right heart, potentially inducing fatal heart failure. SSA in long-acting release may relieve symptoms in more than 50% of patients and reduce systemic serotonin load; however, most patients experience tachyphylaxis and recurrence of particularly diarrhoea may crucially impact QOL [16]. So far treatment for patients with this “refractory” carcinoid syndrome was restricted to pure symptomatic strategies such as opioid-receptor antagonist loperamide or effective tumour debulking by surgery or PRRT. Recently telotristat ethyl, a first-in-class tryptophan hydroxylase inhibitor has been introduced and reduced bowel frequency by ≥30% in 44% of patients in the corresponding TELESTAR trial [17]. In addition, a significant reduction of mean 5‑hydroxyindolyacetic acid (5HIAA) urine levels was documented. Currently telotristat ethyl is available for refractory carcinoid syndrome diarrhoea in a dosage of three times 250 mg daily in combination with SSA analogues.

## Our burning issues in NET

With above discussed approved drugs being available, there is now a relatively wide armamentarium of treatment options available. However, there are several open questions, foremost a potential treatment algorithm for the new G3 NET category. While the combination of temozolomide and capecitabine as presented at this year’s ASCO in Chicago (E211 trial) for pancreatic NET may constitute a good approach, there are currently no prospective data to back decision-making [18]. Furthermore, despite increasing data on the molecular background and genetic landscape of NET including also first data on immunotherapeutic targets, i. e. PD-L1 expression and tumour mutational burden, only very limited clinical data on the value of immunotherapy for NEN exist. In the Keynote-028 study including a cohort of 41 patients with refractory G1/G2 GEP- and lung-NET (PD-L1 positivity ≥1%) preliminary results of median PFS 5.6 months (3.5–10.7) for carcinoid and 4.5 (3.6–8.3) in the pNET cohort were documented [19]. Remarkably, PD-L1 expression appears to increase in poorly differentiated NEC, thus suggesting that this treatment might be interesting for this subgroup facing a particularly poor prognosis [20]. Further data need to be awaited.
